# Did Cannabis Precipitate an STEMI in a Young Woman?

**DOI:** 10.14740/cr398w

**Published:** 2015-06-11

**Authors:** Waqas Jehangir, Michael Stanton, Rafay Khan, Puneet Sahgal, Abdalla Yousif

**Affiliations:** a Raritan Bay Medical Center, USA; b Ross University School of Medicine, Dominica

**Keywords:** Cannabis, Acute coronary syndrome, ST-segment elevation myocardial infarction

## Abstract

Cannabis is a substance that contains compounds that bind cannabinoid receptors, CB1 and CB2. Cannabis also contains substances that do not bind these receptors. Delta-9-tetrahydrocannabinol (THC) is the compound in cannabis responsible for its psychoactive effects and binding to cannabinoid receptors. Despite increasing popularity of the medical and recreational uses of cannabis, little attention has been paid to the adverse effects of the use of the substance. Evidence demonstrating an association between cannabis use and acute coronary syndromes has emerged with case reports and *in vitro* studies. This case report highlights an ST-segment myocardial infarction in a 27-year-old female with little cardiovascular risk factors, but a significant history of frequent cannabis use.

## Introduction

Marijuana has been approved for medicinal use in 21 states following clinical studies demonstrating medical benefits of the drug. In the 1999 issue of the Harvard Mental Health Letter, authors concluded that healthcare professionals view cannabis as a legitimate alternative medication for use in disorders such as neuropathic pain, AIDS wasting syndrome, and chemotherapy-induced emesis. In 2007, a placebo-controlled trial demonstrated that cannabis was effective in relieving neuropathic pain in patients with HIV-associated sensory neuropathy. In 2014, Colorado declared the substance legal for recreational use, after increasing public acceptance of the use of the drug [1]. Despite the increasing acceptance of cannabis for medical and recreational purposes, little is known about the potential adverse effects of the drug, particularly cardiovascular effects. The purpose of this case report is to highlight a particular instance of an acute myocardial infarction in a 27-year-old female with little cardiovascular risk factors, whose toxicology screen was positive only for marijuana.

## Case Report

**Figure 1:**
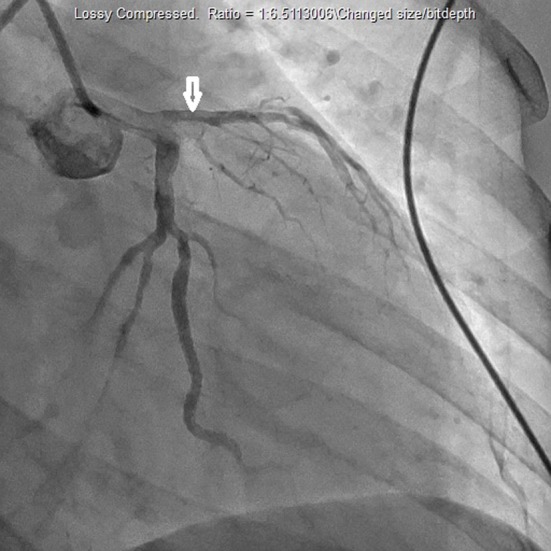
Cardiac catheterization which demonstrated blockage of the left anterior descending artery.

**Figure 2:**
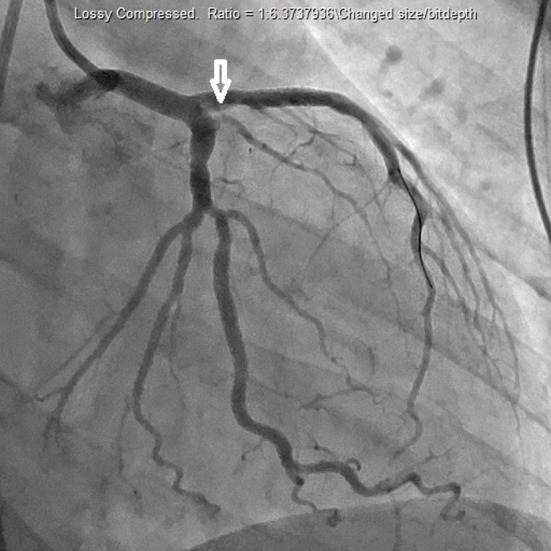
Cardiac catheterization demonstrating revascularization of left anterior descending artery.

A 27-year-old African American female presented to the hospital after a sudden onset of severe retrosternal chest pain. Past medical history was insignificant for hypertension or diabetes mellitus. Patient has a history of smoking cigarettes, beginning at age 9, and frequent marijuana use. The patient was treated with morphine and Percocet for pain. Lab data and urine toxicology screen were negative for troponins and significant for marijuana, respectively. Urine drug screen was negative for cocaine and amphetamines. Drug screen was positive for opiates, but this result is most likely an artifact of morphine and Percocet treatment for chest pain. Initial laboratory findings included hemoglobin 12 g/dL, hematocrit 12.6%, platelets 223 × 10^3^/μL, glucose 156 mg/dL, BUN 9 mg/dL, creatinine 0.8 mg/dL, sodium 135 mEq/L, potassium 4 mEq/L, chloride 101 mEq/L, and bicarbonate 18 mEq/L. In the emergency room, vital signs demonstrated a pulse rate of 98 beats/min, respiratory rate 22 breaths/min, blood pressure 132/89 mm Hg, temperature 98.1 °F, and oxygen saturation 100% on room air. The remainder of the physical examination was unremarkable. Initial electrocardiogram (ECG) demonstrated normal sinus rhythm, and the patient was admitted to telemetry for observation. The subsequent morning, the patient actively experienced chest pain and ECG changes. ECG demonstrated ST segment elevations in leads II and III, and T-wave inversions in V_1_-V_3_. Stat troponin level was found to be elevated at 11.07 ng/mL (< 0.30 ng/mL). The patient immediately underwent cardiac catheterization which demonstrated a 99% blockage of the left anterior descending artery and revascularization was performed (Fig. 1, 2). Two-dimensional echocardiogram demonstrated a left ventricular ejection fraction of 40% with anterior wall akinesis. Transesophageal echocardiogram ruled out an apical mural thrombus, but confirmed the ejection fraction and wall akinesis from the previous echocardiogram. The patient was also found to have hyperlipidemia after the conduction of a lipid panel. Hypercoaguable state and vasculitis workup was negative.

## Discussion

Although cannabis has been advocated for medicinal use in chronic pain syndromes, AIDS wasting syndrome, and chemotherapy-induced emesis, its adverse cardiovascular effects have received only marginal public attention. The purpose of this case report was to highlight an instance of myocardial infarction precipitated by cannabis use in a young 27-year-old female with little cardiovascular risk factors. An *in vitro* study conducted by Deusch et al demonstrated that delta-9-tetrahyrdocannabinol (THC), the psychoactive ingredient of cannabis, significantly increased activation of human platelets via increasing expression of glycoprotein IIb-IIIa and P-selectin [2]. In the same study, western blots demonstrated the expression of cannabinoid receptors on human platelets. The results of the study suggest that the presence of THC in blood may play a role in promoting a prothrombotic environment leading to the precipitation of cardiovascular events. In 2001, Mittleman et al conducted a case-crossover study demonstrating a 4.8-fold increase in risk of an acute myocardial infarction within the first hour after smoking marijuana [3]. Several case reports have emerged over recent years demonstrating an association between cannabis use and cardiovascular events. In 2005, Bachs and Morland reported six cases in which recent cannabis use was linked with sudden, unexpected cardiovascular events leading to death [4]. Cannabis use has been demonstrated to precipitate cardiovascular events in patients with previous cardiovascular disease. Lindsay et al presented two case reports in which one patient with long standing coronary artery disease experienced a malignant arrhythmia after cannabis use [5]. The same study also demonstrated a 22-year-old male, with previously unrecognized coronary artery disease, that experienced an acute myocardial infarction following cannabis use.

Due to increasing support of the use of cannabis for medical and recreational purposes, it has become increasingly important to understand not only the medical benefits, but also the adverse consequences of its administration. This case report is another piece of evidence among others that is highlighting the potential cardiovascular effects of cannabis. Several case reports have emerged in recent years that demonstrate cannabis-induced cardiovascular events, such as myocardial infarction, arrhythmias, and sudden cardiovascular death. *In vitro* studies investigating the role of THC in platelet activation have demonstrated a potential pathophysiological insight into the role of cannabis in cardiovascular events. Further investigations into the association between cannabis use and cardiovascular events are warranted in order to fully appreciate the consequences of cannabis usage, both medical and recreational.
